# Contractile properties and movement behaviour in neonatal rats with axotomy, treated with the NMDA antagonist DAP5

**DOI:** 10.1186/1472-6793-12-5

**Published:** 2012-07-12

**Authors:** Konstantinos Petsanis, Athanasios Chatzisotiriou, Dorothea Kapoukranidou, Constantina Simeonidou, Dimitrios Kouvelas, Maria Albani

**Affiliations:** 1 Department of Physiology, Faculty of Medicine, Aristotle University of Thessaloniki, Thessaloniki, Greece; 2 Department of Experimental Physiology, Faculty of Medicine, Aristotle University of Thessaloniki, Thessaloniki, Greece; 3 2nd Department of Pharmacology, Faculty of Medicine, Aristotle University of Thessaloniki, Thessaloniki, Greece

## Abstract

**Background:**

It is well known that axotomy in the neonatal period causes massive loss of motoneurons, which is reflected in the reduction of the number of motor units and the alteration in muscle properties. This type of neuronal death is attributed to the excessive activation of the ionotropic glutamate receptors (glutamate excitotoxicity). In the present study we investigated the effect of the NMDA antagonist DAP5 [D-2-amino-5-phosphonopentanoic acid] in systemic administration, on muscle properties and on behavioural aspects following peripheral nerve injury.

**Methods:**

Wistar rats were subjected to sciatic nerve crush on the second postnatal day. Four experimental groups were included in this study: a) controls (injection of 0.9% NaCl solution) b) crush c) DAP5 treated and d) crush and DAP5 treated. Animals were examined with isometric tension recordings of the fast extensor digitorum longus and the slow soleus muscles, as well as with locomotor tests at four time points, at P14, P21, P28 and adulthood (2 months).

**Results:**

1. Administration of DAP5 alone provoked no apparent adverse effects. 2. In all age groups, animals with crush developed significantly less tension than the controls in both muscles and had a worse performance in locomotor tests (p < 0.01). Crush animals injected with DAP5 were definitely improved as their tension recordings and their locomotor behaviour were significantly improved compared to axotomized ones (p < 0.01). 3. The time course of soleus contraction was not altered by axotomy and the muscle remained slow-contracting in all developmental stages in all experimental groups. EDL, on the other hand, became slower after the crush (p < 0.05). DAP5 administration restored the contraction velocity, even up to the level of control animals 4. Following crush, EDL becomes fatigue resistant after P21 (p < 0.01). Soleus, on the other hand, becomes less fatigue resistant. DAP5 restored the profile in both muscles.

**Conclusions:**

Our results confirm that contractile properties and locomotor behaviour of animals are severely affected by axotomy, with a differential impact on fast contracting muscles. Administration of DAP5 reverses these devastating effects, without any observable side-effects. This agent could possibly show a therapeutic potential in other models of excitotoxic injury as well.

## Background

Peripheral nerve injury during the critical period of development imparts severe structural and functional consequences on the muscles of the growing animal. It has been well documented that axotomy in the early postnatal period reduces the number of surviving motoneurons in the ventral horn of the lumbar segments and induces changes in the contractile properties of limb muscles [1,2]. These consequences have been ascribed to the critical dependency of the developing motoneurons on their interaction with their target muscle [3,4], as well as to their increased susceptibility to the excitotoxic effects of glutamate [5,6].

Glutamate is the major excitatory neurotransmitter in the CNS. Ionotropic receptors of glutamate (NMDA and AMPA/kainate) have been identified throughout the brain and the spinal cord. In case of overactivation of these receptors, the excessive Ca^2+^ influx into the cell induces a cell death cascade, which comprises the activation of proteases, lipases and other enzymes leading to cell lysis [7]. As it has been shown by previous studies [8-10], this is a time-dependent process, as motoneurons are particularly vulnerable to excitotoxic cell death, only during the first five days of postnatal life.

In the present study we performed sciatic nerve crush in neonatal rats and we investigated the effect of the NMDA antagonist DAP5 [D-2-amino-5-phosphonopentanoic acid] in systemic administration, on muscle properties and on behavioural aspects following injury. This agent has been largely implemented for its antinociceptive action [11-13], as well as for its effects on memory consolidation and hippocampal rhythm [14,15]. In all these studies, the above agent was either delivered intrathecally, or in ex vivo experiments. Systemic application of NMDA receptor antagonists is usually restricted, due to serious side-effects [16,17]. This is the first time, to our best knowledge, that DAP5 has been administered systemically. Our goal was to evaluate both the drug effective dose and its effect on locomotor behaviour and muscular properties.

## Methods

All procedures were performed in accordance with institutional guidelines for the use and care of animals (86/609/EEC) and the ‘Principles of Laboratory animal care’ (NIH publication No 85–23, revised 1985) and were approved by the Ethical Committee for animal experimentation of the Medical School of Thessaloniki (2-3-2006). One hundred seven Wistar rats of both sexes were used in this study. The animals were provided with ad libitum access to food and water and housed in standard cages in a 22°C environment with a 12:12-h light–dark cycle. All efforts were made to minimize the number of animals and their suffering in the experiments.

The pups (N = 80) were divided into four different groups. Unoperated littermates either received DAP5 (N = 20) or remained as untreated controls (injected with normal saline, N = 20). The third experimental group (N = 20) comprised animals subjected to nerve crush and treated with vehicle, whereas in the fourth group (N = 20) were those animals with nerve crush, which underwent treatment with DAP5. The study was performed in four stages of postnatal development (5 animals per age group), on postnatal days, 14, 21, 28 and during adulthood (2 months). The twenty seven remaining rats participated in the titration study.

### Surgical procedures

#### Nerve crush

Adequate anesthesia was initiated and maintained by ether inhalation. Surgery was performed under an operating stereoscope. On the second postnatal day, a small incision was performed in the posterior surface of the left mid-thigh and the sciatic nerve was identified. The crush was performed just proximal to its division to the peroneal and tibial nerve. Attention was paid to avoid damage in the nerve muscular surroundings. Crush was performed by means of a fine pair of forceps which was tightly applied for 30 seconds. Afterwards, the nerve was examined to ensure that the epineural sheath was intact, though translucent. Bleeding was controlled with haemostatic cellulose and the wounds were sutured with 6–0 silk threads. All procedures were carried out by the same researcher. Three hours after recovery from anaesthesia, peanut oil was applied to the wound (to avoid autophagia) and the pups were returned to their mother.

In order to confirm the efficacy of the procedure, the plantar and dorsiflexion reflexes, as well as the inability of normal movement of the left hind limb, with animals suspended from their tail, were assessed daily, for the first 7 days, as described elsewhere [1]. Only animals with verified successful axotomy were included in our study.

### Drug titration-administration

9 groups, each consisting of 3 animals were tested for each dose. Under ether anesthesia, the animals were injected subcutaneously at the interscapular region. The injection was performed daily from P2 to P13. The dose of 50 mg/kg was lethal 24 hours after treatment for all animals. Escalating doses of 10, 20, 30, 40 mg/kg resulted in no changes in rat growth, eating, water drinking, and weight gaining. Doses of 45 mg/kg resulted lethal after 3 days. Doses ranging from 42-45 mg/kg resulted in weight loss or were lethal. At the end, 40 mg/kg was chosen as the treatment dose.

#### Tension recordings

All animals were examined for the contractile properties of two hind limb muscles, the extensor digitorum longus (EDL) and soleus, which represent a fast contracting, easily fatigable and a slow, fatigue resistant muscle, respectively. Animals were anaesthetized with chloral hydrate (4.5%, 10 μl/g body weight, i.p.). The sciatic nerve was identified and prepared proximal to its division. Indifferent to the examined muscle branches of the sciatic nerve were cut. The distal tendons were dissected from the surrounding tissues, cut at their insertion at the bone and attached to a strain gauge transducer (Dynamometer UFI, Devices) by a short silk suture and the exposed parts of the muscles were kept moist with warm (37°C) Krebs-Henseleit solution. (NaCl 118.08 mM, NaHCO_3_ 25 mM, glucose 5.55 mM and CaCl_2_ 1.89 mM). Two pins were inserted in the femoral and calcaneus condyles, thereby adjusting the leg in a position of 90^o^ flexion of the knee and the ankle joints. Muscle length was appropriately adjusted in order to produce maximal single twitch tension (optimal length), through a micromanipulator allowing motion on the 3 axes (Prior, England). The tension elicited by sciatic nerve stimulation (Digitimer DS9A stimulator) was displayed on the monitor using a specific Micro 1501 CED (Cambridge Instruments, UK), after amplification by a DC transducer amplifier (Neurolog NL 107).

Stimulus intensity was adjusted in order to elicit maximal tension, using supramaximal (3–9 volts) square pulses each of 0.5 msec duration. Time to peak (TTP) was calculated by measuring the time taken to reach maximum twitch tension. Time to half relaxation (1/2 RT) was calculated as the time taken for peak twitch tension to decrease to half its original value.

Tetanic contractions were then elicited by stimulating the nerve at 10, 20, 40, 80 and 100 Hz. All devices during the tension recording procedure were controlled by a pulse programmer (Digitimer D4030). The fatigability of the muscles was tested by stimulating the nerve at 40 Hz for 250 ms per second for 180 seconds. During this process, the recorded muscle tension gradually declined as the muscle fibers one by one were losing their contraction ability. Then the fatigue index was calculated as FI = (Initial tension-tension after 180 min)/Initial tension. After tension recordings were completed, the animals were sacrificed and muscles were excised and weighed.

#### Movement behaviour

Movement behaviour was examined by performing 3 kinds of tests. All tests were performed at the same day (P14, P21, P28, 2 months).

1. The Rotarod test in which a rodent was placed on a rotating treadmill. The speed of rotation was gradually increased at an accelerated speed of 4-40 rpm/min. The animals were placed at the treadmill at which time the individual timers started and the rodent’s ability to remain on the rotating rod was recorded. The test lasted for a maximum of 10 minutes. When the animals fell off the treadmill the timer stopped. The purpose of the Rotarod test is to assess the rodent’s sensorimotor coordination [18,19].

2. Bridging: rats are placed in three different (1, 3 and 5 cm wide) narrow wooden lanes of one meter long. Two parameters were examined; the number of errors in passing the bridge and the gait type measured using a particular scale, ranging from 0 to 5 (corresponding to fluent gait with 2 stops, fluent gait with many stops, fearful but with no stops, fearful with stops, particularly difficult).

3. Footprint analysis: The footprint analysis was performed according to Jeroen et al and Alexander Klein et al [20,21] to evaluate hindlimb walking patterns. Briefly, the rats had to walk on strips of paper through a walk away (1 m long, 6 cm wide). Their hindpaws were dipped in blue fountain pen ink. Three series of at least one stepping cycle per side (four sequential steps) were performed per experimental day. The parameters examined were: stride length (distance between left and right footprints), limb rotation (angle between a virtual line through the third digit and the centre of the palm and a virtual line parallel to the walking direction) and distance between feet (distance between feet of the left and right stepping cycle) were analyzed.

### Statistical analysis

Analysis was performed using SPSS 19.0 software for Windows. Animals of the same age group were compared among the different interventions and animals subjected to the same procedure were compared among the different developmental stages. Nonparametric tests were applied. Kruskal – Wallis procedure was initially used in order to detect differences between groups, following which post-hoc pairwise comparisons were performed, by means of a stepwise, step-down method. Criterion of statistical significance was set at p < 0.05.

## Results

The results are presented in detail in Tables 1, 2, 3, 4, 5.

**Table 1:** EDL tension recordings-comparisons

	**AGE**	**PROCEDURE**			
		**CRUSH**	**DAP5**	**CRUSH-DAP5**	**P-VALUE**
**BODY WEIGHT**	**P14**	-	-	-	0.617
	**P21**	-	-	-	0.205
	**P28**	-	-	-	0.771
	**ADULT**	-	-	-	0.523
**MUSCLE WEIGHT**	**P14**	*	-	-	0.005
	**P21**	*	-	#	0.001
	**P28**	*	-	#	0.001
	**ADULT**	*	-	#	0.001
**TIME TO PEAK**	**P14**	*	-	-	0.011
	**P21**	*	-	-	0.003
	**P28**	*	-	-	0.018
	**ADULT**	*	-	-	0.003
**HALF RELAXATION TIME**	**P14**	*	-	-	0.025
	**P21**	*	-	-	0.003
	**P28**	*	-	-	0.007
	**ADULT**	*	-	-	0.005
**SINGLE TWITCH**	**P14**	*	-	-	0.011
	**P21**	*	-	-	0.003
	**P28**	*	-	#	0.001
	**ADULT**	*	-	#	0.001
**TETANIC-100**	**P14**	*	-	#	0.002
	**P21**	*	-	-	0.003
	**P28**	*	-	#	0.001
	**ADULT**	*	-	#	0.001
**FATIGUE INDEX**	**P14**	-	-	*	0.007
	**P21**	*	-	#	0.003
	**P28**	*	-	-	0.003
	**ADULT**	*	-	-	0.009

Post-hoc multiple comparisons. The actual parameters are presented in the graphs. *: different from control. ^#^: different from crush. Level of significance was a = 0.05.

**Table 2:** EDL tension recordings

**EDL**						
	**AGE**	**PROCEDURE**				**ANOVA ON THE RANKS**
					**CONTROL**	**CRUSH**	**DAP5**	**CRUSH-DAP5**	**P-VALUE**
**WEIGHT-INDEX**	**P14**	0.04 ± 0.01	0.02 ± 0.^*^	0.04 ± 0.01	0.03 ± 0.01	0.017
	**P21**	0.05 ± 0.01	0.02 ± 0.^*^	0.04 ± 0.01	0.03 ± 0.^#^	0.002
	**P28**	0.06 ± 0.01	0.02 ± 0.^*^	0.06 ± 0.01	0.04 ± 0.^#^	0.001
	**ADULT**	0.08 ± 0.01	0.01 ± 0.^*^	0.09 ± 0.01	0.08 ± 0.01	0.003
**TETANIC-10 (g)**	**P14**	6.59 ± 0.89	1.94 ± 0.16^*^	7.21 ± 1.21	6.79 ± 0.29	0.011
	**P21**	9.96 ± 0.63	4.68 ± 0.79^*^	10.66 ± 1.37	9.39 ± 1.71	0.010
	**P28**	17.4 ± 1.39	4.94 ± 0.26^*^	18.24 ± 0.53	15.05 ± 1.95^#^	0.002
	**ADULT**	86.72 ± 3.55	6.05 ± 0.63^*^	87.42 ± 4.1	72.59 ± 2.85^#^	0.001
**TETANIC-20 (g)**	**P14**	8.13 ± 1.3	2.5 ± 0.34^*^	8.06 ± 1.02	7.62 ± 0.46	0.012
	**P21**	13.27 ± 1.52	6.04 ± 0.64^*^	12.51 ± 0.86	10.65 ± 1.54	0.003
	**P28**	24.51 ± 1.19	5.6 ± 0.41^*^	24.45 ± 0.98	20.88 ± 1.68^#^	0.001
	**ADULT**	117.45 ± 5.37	7.71 ± 0.96^*^	119.52 ± 4.78	105.08 ± 13.66	0.004
**TETANIC-40 (g)**	**P14**	9.46 ± 1.64	3.3 ± 0.48^*^	9.79 ± 1.65	8.87 ± 0.46	0.011
	**P21**	18.46 ± 1.06	8.15 ± 0.37^*^	17.53 ± 0.91	14.61 ± 1.44^#^	0.001
	**P28**	37.92 ± 1.44	8.9 ± 0.58^*^	38.75 ± 1.66	31.48 ± 2.91^#^	0.001
	**ADULT**	177.78 ± 14.95	13.22 ± 1.15^*^	179.26 ± 8.94	140.83 ± 10.29^#^	0.001
**TETANIC-80 (g)**	**P14**	10.35 ± 1.78	3.91 ± 0.52^*^	10.63 ± 1.82	9.57 ± 0.32	0.012
	**P21**	22.42 ± 1.22	10.87 ± 1.29^*^	21.36 ± 0.96	17.49 ± 1.94^#^	0.001
	**P28**	49.95 ± 1.34	10.79 ± 0.97^*^	52.9 ± 1.06	41.66 ± 1.97^#^	0.001
	**ADULT**	208.5 ± 8.55	14.43 ± 0.88^*^	205.65 ± 6.95	164.84 ± 11.33^#^	0.001
**FORCE/WEIGHT (g/g)**	**P14**	1559.86 ± 292.25	834.2 ± 150.66^*^	1379.97 ± 404.56	1565.73 ± 367.12	0.018
	**P21**	1021.27 ± 308.81	1474.97 ± 340.35^*^	1081.54 ± 86.26	1390.81 ± 306.78^*^	0.034
	**P28**	1124.87 ± 156.98	571.16 ± 89.02^*^	1099.9 ± 152.2	1247.61 ± 53.81	0.006
	**ADULT**	1460.03 ± 89.47	792.95 ± 138.6^*^	1407.11 ± 44.59	1341.69 ± 81.63	0.005

Values are expressed as Mean ± Standard deviation. Post-hoc multiple comparisons. *: different from control. ^#^: different from crush. Level of significance was a = 0.05.

**Table 3:** Soleus recordings-comparisons

	**AGE**	**PROCEDURE**			
		**CRUSH**	**DAP5**	**CRUSH-DAP5**	**P-VALUE**
**BODY WEIGHT**	**P14**	-	-	-	0.891
	**P21**	-	-	-	0.064
	**P28**	-	-	-	0.918
	**ADULT**	-	-	-	0.944
**MUSCLE WEIGHT**	**P14**	-	-	-	0.257
	**P21**	*	-	-	0.006
	**P28**	*	-	#	0.001
	**ADULT**	*	-	#	0.001
**TIME TO PEAK**	**P14**	-	-	-	0.630
	**P21**	-	*	-	0.007
	**P28**	*	-	-	0.048
	**ADULT**	-	-	-	0.744
**HALF RELAXATION TIME**	**P14**	-	-	-	0.447
	**P21**	-	*	-	0.009
	**P28**	-	-	-	0.219
	**ADULT**	-	-	*	0.009
**SINGLE TWITCH**	**P14**	*	-	-	0.005
	**P21**	*	-	-	0.036
	**P28**	*	-	#	0.002
	**ADULT**	*	-	#	0.002
**TETANIC-100**	**P14**	*	-	-	0.003
	**P21**	*	-	-	0.023
	**P28**	*	#	#	0.001
	**ADULT**	*	-	#	0.001
**FATIGUE INDEX**	**P14**	*	-	-	0.009
	**P21**	*	-	-	0.031
	**P28**	-	-	-	0.063
	**ADULT**	*	-	-	0.010

Post-hoc multiple comparisons. The actual parameters are presented in the graphs. *: different from control. ^#^: different from crush. Level of significance was a = 0.05.

**Table 4:** Soleus tension recordings

**SOLEUS**						
	**AGE**	**PROCEDURE**				**ANOVA ON THE RANKS**
		**CONTROL**	**CRUSH**	**DAP5**	**CRUSH-DAP5**	**P-VALUE**
**WEIGHT-INDEX**	**P14**	0.03 ± 0.	0.03 ± 0.	0.03 ± 0.01	0.03 ± 0.01	0.364
	**P21**	0.03 ± 0.	0.03 ± 0.^*^	0.03 ± 0.01	0.03 ± 0.	0.039
	**P28**	0.04 ± 0.	0.02 ± 0.^*^	0.04 ± 0.	0.03 ± 0.^#^	0.001
	**ADULT**	0.06 ± 0.	0.01 ± 0.^*^	0.06 ± 0.	0.04 ± 0.^#^	0.001
**TETANIC-10 (g)**	**P14**	5.85 ± 1.35	1.65 ± 0.18^*^	5.06 ± 0.59	4.64 ± 0.67	0.005
	**P21**	11.99 ± 0.68	9.88 ± 1.02^*^	13.84 ± 1.26^#^	13.3 ± 0.69^#^	0.005
	**P28**	21.43 ± 2.06	10.92 ± 1.04^*^	20.1 ± 1.83	18.39 ± 2.03	0.004
	**ADULT**	44.65 ± 0.81	9.15 ± 0.59^*^	41.72 ± 2.27^#^	40.77 ± 2.31^#^	0.002
**TETANIC-20 (g)**	**P14**	6.78 ± 1.29	2.47 ± 0.44^*^	6.59 ± 0.75	5.45 ± 0.74	0.003
	**P21**	16.94 ± 0.97	14.49 ± 0.74^*^	14.73 ± 1.51^*^	14.25 ± 0.9^*^	0.020
	**P28**	35.76 ± 2.16	15.44 ± 0.6^*^	27.75 ± 2.08^#^	26.39 ± 2.57^#^	0.001
	**ADULT**	54.1 ± 0.92	10.62 ± 0.89^*^	53.01 ± 1.65	49.61 ± 2.56^#^	0.002
**TETANIC-40 (g)**	**P14**	8.46 ± 1.65	2.84 ± 0.3^*^	9.98 ± 0.73	8.05 ± 0.83	0.003
	**P21**	24.27 ± 0.98	19.96 ± 2.02^*^	23.76 ± 1.39	21.71 ± 1.34^#^	0.007
	**P28**	52.11 ± 2.51	17.63 ± 2.33^*^	32.62 ± 2.99^#^	29.19 ± 1.82^#^	0.001
	**ADULT**	89.15 ± 7.15	14.16 ± 1.22^*^	91.78 ± 6.08	80.85 ± 3.04^#^	0.002
**TETANIC-80 (g)**	**P14**	10.65 ± 1.32	3.48 ± 0.37^*^	11.91 ± 0.96	9.54 ± 0.65^#^	0.002
	**P21**	33.56 ± 1.09	29.96 ± 1.7^*^	34.41 ± 2.25	32.47 ± 1.66	0.013
	**P28**	69.89 ± 1.69	20.71 ± 2.92^*^	40.38 ± 2.1^#^	37.79 ± 1.83^#^	0.001
	**ADULT**	130.03 ± 1.92	16.89 ± 0.98^*^	129.45 ± 2.36	121.16 ± 5.06^#^	0.002
**FORCE/WEIGHT (g/g)**	**P14**	2037.05 ± 529.05	787.3 ± 156.^*^	2534.11 ± 461.66	1648.11 ± 392.76	0.003
	**P21**	2046.25 ± 187.04	2370.38 ± 252.19^*^	1980.12 ± 357.46	2225.41 ± 213.59	0.131
	**P28**	2710.54 ± 134.72	1272.12 ± 108.09	1421.89 ± 223.69^#^	1687.99 ± 175.58^#^	0.001
	**ADULT**	1111.12 ± 25.62	787.93 ± 195.12^*^	1104.13 ± 20.91	1591.63 ± 127.07^#^	0.001

Values are expressed as Mean ± Standard deviation. Post-hoc multiple comparisons. *: different from control. ^#^: different from crush. Level of significance was a = 0.05.

**Table 5:** Locomotor tests

	**AGE**	**PROCEDURE**	**ANOVA ON THE RANKS**			
		**CONTROL**	**CRUSH**	**DAP5**	**CRUSH-DAP5**	**P-VALUE**
**Rotarod (sec)**	**P14**	264.20 ± 12.28	8.40 ± 2.70*	256.00 ± 12.02	19.00 ± 3.39 ^#^	0.001
	**P21**	289.80 ± 14.94	20.40 ± 6.50^*^	276.40 ± 16.02	51.40 ± 7.86^#^	0.001
	**P28**	289.20 ± 11.80	37.20 ± 9.15^*^	290.60 ± 12.92	104.20 ± 9.98^#^	0.001
	**ADULT**	285.80 ± 13.57	45.80 ± 7.98^*^	289.40 ± 15.03	229.80 ± 12.34^#^	0.001
**Bridge 1 cm**	**P14**	1.20 ± 0.45	8.60 ± 0.55^*^	1.00 ± 0.71	4.20 ± 0.84^#^	0.001
	**P21**	0.80 ± 0.84	7.40 ± 0.89^*^	0.60 ± 0.55	2.60 ± 1.52	0.003
	**P28**	0.80 ± 0.84	7.20 ± 0.84^*^	1.00 ± 0.71	2.80 ± 0.45^#^	0.001
	**ADULT**	0.80 ± 0.45	6.20 ± 1.10^*^	0.80 ± 0.45	3.00 ± 0.71^#^	0.001
**Bridge 3 cm**	**P14**	0.80 ± 0.84	7.60 ± 0.89^*^	0.60 ± 0.55	2.80 ± 0.84^#^	0.001
	**P21**	0.40 ± 0.89	7.00 ± 0.71^*^	0.40 ± 0.55	2.80 ± 0.84^#^	0.001
	**P28**	0.60 ± 0.55	5.80 ± 1.30^*^	0.60 ± 0.89	2.20 ± 0.84^#^	0.002
	**ADULT**	0.20 ± 0.45	5.00 ± 1.58^*^	0.60 ± 0.55	2.20 ± 0.84^#^	0.001
**Bridge 5 cm**	**P14**	0.60 ± 0.55	6.20 ± 0.45^*^	0.00 ± 0.00	2.40 ± 0.55^#^	0.001
	**P21**	0.40 ± 0.55	4.40 ± 1.14^*^	0.40 ± 0.55	1.60 ± 1.14	0.004
	**P28**	0.40 ± 0.55	4.40 ± 1.14^*^	0.20 ± 0.45	2.00 ± 1.00^#^	0.001
	**ADULT**	0.00 ± 0.00	2.80 ± 0.84^*^	0.20 ± 0.45	1.80 ± 0.84^*^	0.001
**Gait 1 cm**	**P14**	2.80 ± 0.84	4.60 ± 0.89^*^	2.60 ± 1.14	3.40 ± 0.89	0.037
**(errors)**	**P21**	2.40 ± 1.14	4.60 ± 0.55^*^	2.20 ± 1.48	3.00 ± 0.71	0.016
	**P28**	2.80 ± 0.84	3.80 ± 0.84	2.40 ± 1.14	2.60 ± 0.89	0.149
	**ADULT**	2.40 ± 1.14	4.00 ± 1.00	2.00 ± 1.58	2.00 ± 1.22	0.091
**Gait 3 cm**	**P14**	2.40 ± 0.89	4.00 ± 0.71^*^	2.20 ± 0.84	2.80 ± 1.10	0.025
**(errors)**	**P21**	1.80 ± 0.84	3.80 ± 0.84^*^	1.80 ± 1.30	1.80 ± 1.30	0.036
	**P28**	1.80 ± 1.48	4.20 ± 0.84^*^	1.60 ± 0.89	2.00 ± 0.00	0.014
	**ADULT**	1.80 ± 1.30	3.80 ± 1.30	1.60 ± 1.14	1.80 ± 1.30	0.094
**Gait 5 cm**	**P14**	2.40 ± 1.52	3.40 ± 0.55	1.60 ± 1.14	3.00 ± 1.00	0.098
**(errors)**	**P21**	1.40 ± 1.14	3.00 ± 0.71^*^	0.80 ± 0.84	1.40 ± 0.55	0.019
	**P28**	1.80 ± 1.48	3.60 ± 0.89^*^	1.80 ± 0.45	1.20 ± 1.30	0.029
	**ADULT**	1.20 ± 1.30	3.40 ± 1.52	1.20 ± 0.84	1.20 ± 0.84	0.089
**Limb Rotation**	**P14**	13.40 ± 0.98	0.00 ± 0.00	13.18 ± 0.76	14.42 ± 0.89	0.194
	**P21**	17.88 ± 0.44	21.24 ± 0.86^*^	17.90 ± 0.53	20.00 ± 0.83^*^	0.002
	**P28**	18.62 ± 1.21	22.56 ± 0.79^*^	18.50 ± 0.86	22.12 ± 0.79^*^	0.002
	**ADULT**	20.76 ± 1.87	27.28 ± 0.56^*^	20.62 ± 1.64	24.60 ± 0.72^#^	0.001
**Stride Length (cm)**	**P14**	3.78 ± 0.53	0.00 ± 0.00	3.70 ± 0.29	2.30 ± 0.51^*^	0.010
	**P21**	11.86 ± 0.56	9.94 ± 0.44^*^	12.02 ± 0.57	10.10 ± 0.70^*^	0.003
	**P28**	15.78 ± 1.18	13.56 ± 0.41^*^	15.42 ± 1.19	13.76 ± 0.60^*^	0.006
	**ADULT**	17.82 ± 0.68	13.14 ± 0.95^*^	18.02 ± 0.51	16.44 ± 0.54^#^	0.001
**DBF(cm)**	**P14**	0.98 ± 0.18	0.00 ± 0.00	0.98 ± 0.22	0.64 ± 0.13^*^	0.027
	**P21**	1.86 ± 0.18	1.30 ± 0.19^*^	2.02 ± 0.20	1.36 ± 0.13^*^	0.002
	**P28**	2.70 ± 0.19	2.96 ± 0.11	2.78 ± 0.16	2.20 ± 0.14^*^	0.003
	**ADULT**	3.34 ± 0.23	3.80 ± 0.19^*^	3.43 ± 0.19	2.92 ± 0.16^#^	0.003

Values are expressed as Mean ± Standard deviation. Post-hoc multiple comparisons. *: different from control. ^#^: different from crush. Level of significance was a = 0.05.

### A. Body-weight-muscle weight

**Figure 1:**
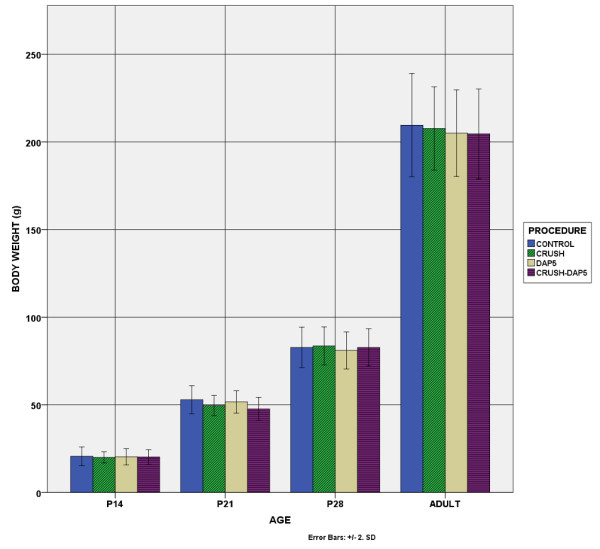
**Body Weight.** No statistical significance was found among the experimental groups. Body weight increased with age in all groups.

**Figure 2:**
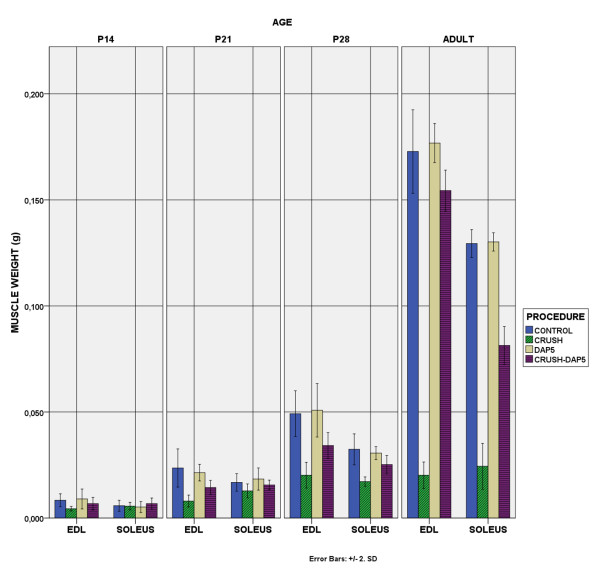
**Muscle Weight.** Muscle weight was reduced by crush, after P14 in EDL and after P21 in soleus (p < 0.01). DAP-5 increased muscle weight, but not to the level of control animals.

Body weight did not differ between the experimental groups (Figure 1). Muscle weight (Figure 2) and weight index (muscle/body weight) in crush animals was definitely reduced compared to controls (p < 0.01). This reduction was already apparent by P14 in EDL, whereas in soleus was evident after P21. Similarly, in crush animals which received DAP5, the difference compared to controls was evident after P14 in EDL and after P21 in soleus. These animals also differed from animals with crush, with differences becoming evident earlier, already at P14 in EDL and at P21 in soleus. We have to note that during normal development weight index changed (increased in adulthood compared to P14, p < 0.01). Axotomy progressively reduced the index, presumably due to muscle weight decrease. DAP-5 reversed this situation for both muscles.

### B. Isometric tension recordings

**Figure 3:**
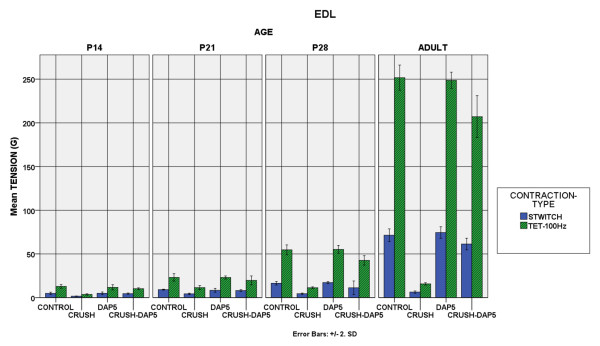
**EDL Force Generation.** Bar chart showing the contractile force of the EDL muscle. Single twitch and maximum tetanic contraction at 100 Hz is depicted for all experimental groups in all ages. It is evident that crush animals developed significantly less tension than the controls and DAP5 administration clearly improved muscle performance.

**Figure 4:**
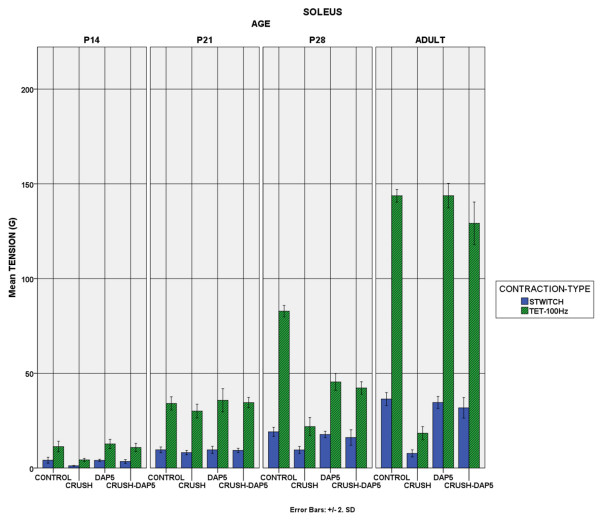
**Soleus Force Generation.** Similar to Figure 1, showing the contractile force of soleus muscle.

Tension development: Treatment with DAP5 did not alter the normal muscle properties, the only exception being the soleus muscle in P21 and P28 animals, in which the tetanic tensions were unexpectedly lower (p < 0.01). The absence of muscle “side effects” was consistent for both muscles in all age groups. In adult rats, EDL single twitch in controls, DAP5–treated, crush and crush + DAP5 treatment was 71.37 ± 3.64, 74.43 ± 3.30, 6.27 ± 0.67 and 61.24 ± 3.32gr, respectively (values expressed as mean ± SD).When soleus was considered, the respective values were 36.41 ± 1.71, 34.62 ± 1.56, 7.72 ± 0.93 and 31.76 ± 2.71gr. In all age groups, animals with crush exhibited significantly less tension than the controls in both muscles (p < 0.01). Crush animals injected with DAP5 were definitely improved as their tension recordings were significantly higher than the crush ones without DAP5 in both muscles (p < 0.01). This improvement however, did not generally reach the level of control animals, nor those with DAP5 injection (Figure 3–4).

**Figure 5:**
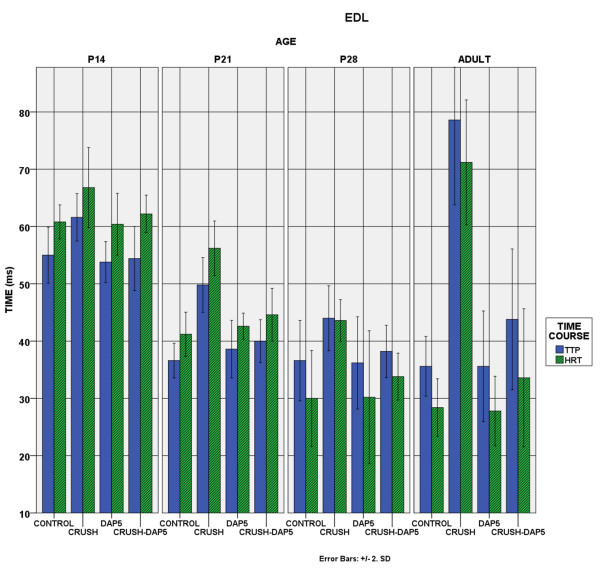
**EDL Time course of Contraction.** Bar chart showing the evolution of the two parameters of the time course of contraction, among the different experimental groups. TTP:time-to-peak and HRT:Half-relaxation-time. Immature EDL is a slow muscle, but progressively its contraction is shortened in order to attain the fast profile of the adult animal. Crush disrupts this process and the muscle remains slow in all developmental stages.

**Figure 6:**
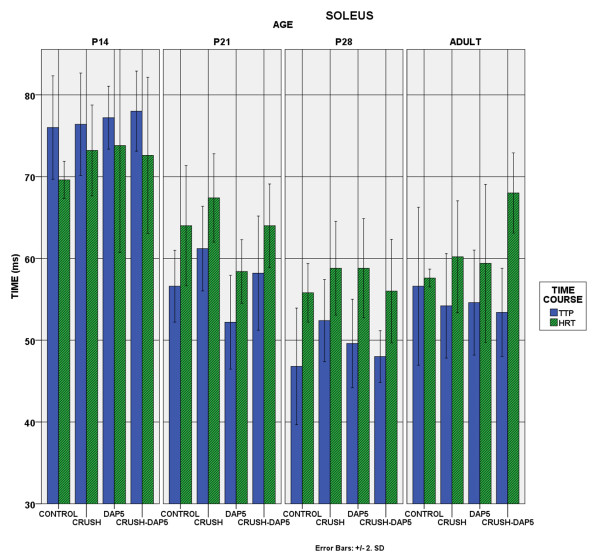
**Soleus Time course of Contraction.** Bar chart showing the evolution of the two parameters of the time course of contraction, among the different experimental groups. TTP:time-to-peak and HRT:Half-relaxation-time. Soleus remains a slow muscle in all developmental stages, in all procedures.

Time course of contraction: The time course of soleus contraction was not altered by axotomy and the muscle remained slow-contracting in all developmental stages, in all experimental groups. EDL, on the other hand, became slower after the crush (p < 0.05). DAP5 administration restored the contraction velocity, up to the level of control animals (Figure 5–6).

**Figure 7:**
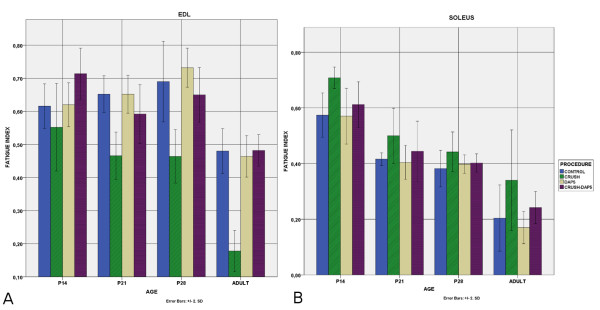
**Fatigue index.** Bar chart showing the fatigue index for both muscles (**A.**EDL **B.**Soleus) in all developmental stages for all procedures. Fatigue index normally decreases by age. Crush renders EDL fatigue resistant after P21, while soleus becomes more fatiguable. DAP5 restored the profile in both muscles.

Fatigue index: Following crush, EDL becomes fatigue resistant after P21. In adult animals with crush the index was 0.18 ± 0.03 vs 0.48 ± 0.03 in controls (p < 0.01). Soleus, on the other hand, becomes less fatigue resistant (0.2 ± 0.06 in controls vs 0.34 ± 0.09 in axotomized adults, p < 0.01). DAP5 administration restored the profile in both muscles (EDL: 0,48 ± 0.02, soleus: 0.24 ± 0.03, p < 0.01 compared to crush), up to the level of control animals (no difference after P28)(Figure  7).

Specific tension: This parameter (tetanic tension 100 Hz/muscle weight) was reduced in crush animals in both muscles (p < 0.01). DAP5 administration reversed this effect.

### C. Movement behaviour

**Figure 8:**
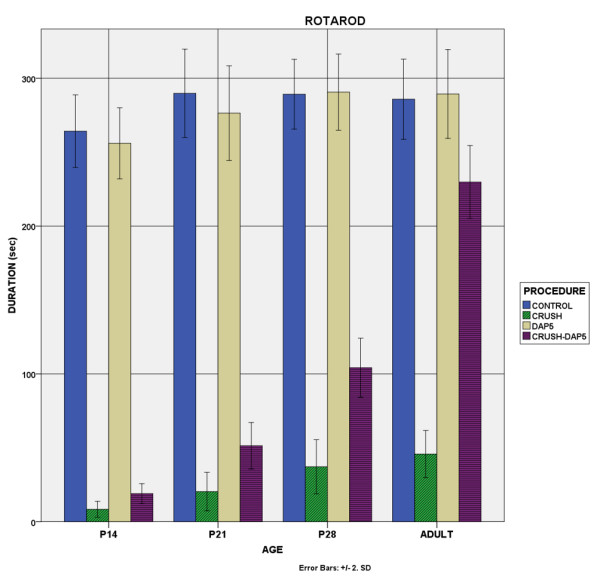
**Rotarod test.** Bar chart showing the duration that the animals achieved on the rotarod. Crush resulted in a definite reduction in time and DAP-5 increased the capacity.

The results are presented in Table 5 and the performance in rotarod test is shown in Figure 8. It should be noticed that P14-crush animals were not able to perform the limb rotation, stride length and DBF, presumably due to their young age to participate in these tests.

Injection of DAP5 had no impact in animal locomotion, as there was no significant difference compared to controls, in any age group, in any parameter studied.

Among the various experimental groups, crush animals had definitely lower motor scores than the controls (p < 0.05). These differences remained throughout all ages, apart from adults, in which the gaits exhibited no significant changes. DAP5 administration in axotomized animals improved motor behaviour (p < 0.05 compared to axotomized). For limb rotation, stride length and DBF, the difference became evident after P28. This improvement, however, reached the level of neither the controls (p < 0.05), nor those with intact nerve and DAP5 treatment (p < 0.05).

Concerning the evolution of locomotor behaviour, the animal performance in the gaits and the bridges did not exhibit any discernible difference, as the animals grew older. On the other hand, the rotarod, the limb rotation, the stride length and the DBF provided a more robust index of the differentiation of the animals’ locomotion, with significant differences between the age groups (p < 0.05).

## Discussion

It is well established that peripheral nerve crush injury, during early postnatal development, results in significant loss of motor neurons and extensive muscle atrophy [1,9]. The mechanism of cell death involves, on the one hand, the activation of several apoptotic pathways [22] and on the other hand the necrotic cell death, probably caused by glutamate-mediated excitotoxicity [2,6]. The differential response between mature and immature motoneurons following injury is attributed to the quantity of glutamate receptors on the cell membrane [23,24].

Administration of an NMDA or AMPA/Kainate receptor antagonist within this critical period of development is thought to reverse the neurotoxic effects of axotomy and result in increased survival of motoneurons [2,5,7,25,26]. Unfortunately, the protective effects for many of these factors are only transient, lasting 2–3 weeks [27]. Dizocilpine malate (MK-801), an NMDA antagonist, has been used in animal models in vivo with success, in order to prevent motoneuron death after axotomy. It was badly tolerated by rats, however, due to side effects and high mortality [2,25]. Furthermore, magnesium, which is known to act as a voltage-dependent blocker of the N-methyl-D-aspartate (NMDA) channel, by coupling with the specific Mg^2+^ site within the pore of the ion channel [28,29], was found to inhibit the death of ventral horn motoneurons and to restore the alteration in contractile properties provoked by axotomy [1].

In the present study we assessed the contractile properties and the movement behaviour in rats of different age groups, following neonatal sciatic nerve crush and administration of DAP5. This is a selective NMDA receptor antagonist that competitively inhibits the ligand (glutamate) binding site of NMDA receptors. DAP5 is generally very fast acting as indicated by in vitro preparations, and can block NMDA receptor action at a reasonably small concentration [30]. Our hypothesis was that, by delivering an agent with a direct action on the NMDA receptor, we would be able to achieve a more profound effect, than the one observed with the indirect action of magnesium. A drawback in our study is that axotomized hindlimbs were not compared with the ones of the opposite side (right), but with those of control animals, thus rendering our observations more vulnerable to interanimal differences. We chose that design, however, to achieve a correlation with the behavioural tests, which necessarily had to entail a control group of animals. Moreover, body weight did not differ among the experimental groups and consequently the differences in tension recordings may be directly ascribed to the muscle changes.

According to our knowledge, this is the first time that DAP5was administered in vivo. Systematic administration of DAP5 has been discouraged by other researchers, due to poor cerebrospinal fluid (CSF) absorption and probable toxic features [17]. By initially following titration trials, we did not observe any side effects. In all age groups, no significant difference was found between control animals and those that the agent was administered, in both contractile properties and behavioural tests. These results allowed us to conclude that a safe and effective therapeutic profile is evident for the aforementioned drug, at least for the parameters studied.

Apart from reducing the number of surviving motoneurons, axotomy in the early postnatal period alters the contractile properties of limb muscles, as well [3]. Our results are in accordance with our previous work [1], as well as other researchers [2], showing that axotomy severely impairs tension development by the muscle. The main feature in this study is that DAP5 resulted in the recovery of the contractile properties of both muscles, up to the level of control animals, thus fully eliminating the debilitating effect of axotomy. We assume that the direct action of the agent on the NMDA receptor accounts for the improved results.

Concerning the time evolution of contraction, we reconfirmed that immature (P14) muscles are not yet differentiated into fast- or slow-contracting ones and that fast contracting muscles are more severely affected by axotomy [1,3,31]. In the early developmental stages, contraction in both muscles is rather prolonged, with a high fatigue index. In control animals, EDL attains its normal features by adulthood, in the case of axotomy, however, the muscle becomes slow and fatigue resistant. At this point, there was a differentiation concerning our previous work, as DAP5 succeeded in recuperating in a greater degree the original features of the muscle. In agreement with the majority of researchers, it was of no surprise that soleus did not present any alteration in its time course of contraction, as its contractile properties are not significantly altered throughout early postnatal life. Concerning fatigability, normally soleus is converted progressively into a fatigue resistant muscle. This process is halted, in case of axotomy and is partially reversed by DAP5 administration. We also have to point out that differences were statistically significant in most parameters among the various age groups (within the same experimental group), reflecting the fact that all animals underwent the natural course of contractile properties maturation (force augmentation during development).

In order to evaluate the locomotion, a series of tests was applied, which comprised the rotarod, as well as passing along bridges of different width and footprint analysis. The results were in full correlation with the isometric recordings. Rats with axotomy exhibited overt changes in their locomotor behaviour, compared with controls. Treatment with DAP5 improved movement, although the difference with controls was still discernible. Improvement in locomotor behaviour, following DAP5 administration, however, was not as impressive as the one observed in tension recordings. Axotomy provokes a serious sensorimotor disruption, early in development, and coordination of the limbs during walking does not entirely rely on the reinforcement of individual muscles. Adaptive mechanisms are activated to compensate for the lack of function; the injured rat exerts a notifying effort to use the crushed leg and eventually succeeds in walking, nevertheless, with uncontestable difference with respect to the non-injured one. In addition, it seems that some tests are more specific in delineating subtle differences between the different groups. The rotarod and the footprint analysis turned out to evaluate locomotor behaviour in a more efficient way, than the observation and the grading of the gait.

The lack of side-effects seems rather unexpected in our study, compared to what has been described in the literature [32]. The NMDA receptor contributes to plasticity, neuronal differentiation and synaptogenesis in the developing nervous system [33]. NMDA antagonists are notorious for causing a multitude of behavioural sequelae. The disfunction of this receptor is frequently considered to contribute to the pathophysiology of schizophrenia, depression, anxiety and, indeed, these agents are usually implemented in the research of several psychotic states [34]. Moreover, MK-801, one of the most extensively studied drugs in this category, is known to induce long term behavioural disturbances, when administered in neonatal rats [35]. One could argue that our locomotor models may not be so sensitive to detect this kind of behavioural defect. Furthermore, despite sharing nominally common mechanisms of action and often presumed biological equivalence, the NMDA antagonists present very diverse effects [36]. Most antagonists used in animal studies, such as MK-801, act via an uncompetitive antagonism, whereas DAP5 utilizes a competitive mode of action. It might be that, this mode of action, in association with the affinity of the receptor, should provide an effective combination which varies among the different substances and explains the magnitude of primary actions as well as the side effects.

## Conclusions

Our results show that contractile properties and locomotor behaviour of animals are severely affected by axotomy, with a differential impact on fast contracting muscles. Administration of DAP5 reverses these devastating effects. To our knowledge, this is the first time that the systematic action of DAP5 is studied and the absence of apparent side-effects is very important, although further research is certainly required, in order to detect direct or indirect, local or systemic actions, which were not identified in our study.

The implications of such findings are apparent. By possessing a relatively safe pharmacologic profile and the encouraging results described above, this agent could be explored in a variety of animal models dealing with excitotoxic cell death.

## Abbreviations

NMDA: N-methy-D-Aspartate; AMPA: A-amino-isoxazolopropionic acid; DAP5: D-2-Amino-5-phosphonopentanoic acid; EDL: Extensor digitorum longus; DBF: Distance between feet.

## Misc

Konstantinos Petsanis and Athanasios Chatzisotiriou contributed equally to this work

## Competing interests

The authors declare that they have no competing interests.

## Authors’ contribution

CP was the primary researcher, was involved in the initial design of the study and contributed to the writing of the manuscript. AC carried out the data analysis and drafted the manuscript. CS has contributed to the design of the behavioural part of the study and helped to draft the manuscript. DK conducted a part of the experimental procedures. DK had a substantial involvement in the pharmacologic design of the study and the performance of the titration procedure. MA conceived of the study and participated in its design, coordination and supervision. All authors read and approved the final manuscript.
